# Fibroblast Growth Factor Signaling in Metabolic Regulation

**DOI:** 10.3389/fendo.2015.00193

**Published:** 2016-01-19

**Authors:** Vera J. M. Nies, Gencer Sancar, Weilin Liu, Tim van Zutphen, Dicky Struik, Ruth T. Yu, Annette R. Atkins, Ronald M. Evans, Johan W. Jonker, Michael Robert Downes

**Affiliations:** ^1^Center for Liver, Digestive and Metabolic Diseases, Department of Pediatrics, University Medical Center Groningen, University of Groningen, Groningen, Netherlands; ^2^Gene Expression Laboratory, Salk Institute for Biological Studies, La Jolla, CA, USA

**Keywords:** fibroblast growth factors, metabolic syndrome, FGF signaling, therapeutic potential, FGF1, FGF19, FGF21

## Abstract

The prevalence of obesity is a growing health problem. Obesity is strongly associated with several comorbidities, such as non-alcoholic fatty liver disease, certain cancers, insulin resistance, and type 2 diabetes, which all reduce life expectancy and life quality. Several drugs have been put forward in order to treat these diseases, but many of them have detrimental side effects. The unexpected role of the family of fibroblast growth factors in the regulation of energy metabolism provides new approaches to the treatment of metabolic diseases and offers a valuable tool to gain more insight into metabolic regulation. The known beneficial effects of FGF19 and FGF21 on metabolism, together with recently discovered similar effects of FGF1 suggest that FGFs and their derivatives carry great potential as novel therapeutics to treat metabolic conditions. To facilitate the development of new therapies with improved targeting and minimal side effects, a better understanding of the molecular mechanism of action of FGFs is needed. In this review, we will discuss what is currently known about the physiological roles of FGF signaling in tissues important for metabolic homeostasis. In addition, we will discuss current concepts regarding their pharmacological properties and effector tissues in the context of metabolic disease. Also, the recent progress in the development of FGF variants will be reviewed. Our goal is to provide a comprehensive overview of the current concepts and consensuses regarding FGF signaling in metabolic health and disease and to provide starting points for the development of FGF-based therapies against metabolic conditions.

## Introduction

Fibroblast growth factors (FGFs) are secreted signaling proteins with wide ranging functions in cell proliferation, development, and wound healing (1, 2). FGFs act as autocrine, paracrine, and/or endocrine hormones by binding to FGF receptors (FGFRs). FGFR dimerization induces the activation of downstream signaling cascades. Over the past two decades, several FGFs have been linked to metabolism by the discovery that they are transcriptionally regulated by members of the nuclear receptor (NR) superfamily of ligand-activated transcription factors (3–8). These FGFs have been demonstrated to mediate some of the effects of these NRs in the regulation of glucose and lipid metabolism (3, 9, 10). Currently, three members have been linked to regulation of energy metabolism: FGF1, FGF15/19 (with FGF15 being the mouse ortholog of human FGF19), and FGF21 (3–6). FGF1 is critical for adipose function and is regulated by the lipid sensor PPARγ, FGF15/19 modulates bile acid metabolism and is regulated by the bile acid sensor farnesoid X receptor (FXR), and FGF21 regulates the adaptive fasting response and is a target of the fatty acid sensor PPARα (3, 9, 10).

FGF15/19 is considered as a regulator of the feeding response. In response to food intake, a postprandial flux of bile acids is released into the small intestine followed by activation of FXR expressed in the terminal ileum, which results in increased transcription of FGF15/19 (9). FGF15/19 enters the circulation and binds to the FGFR4/β-klotho receptor complex on the cell membrane of hepatocytes, ultimately leading to repression of gluconeogenesis and stimulation of glycogen and protein synthesis (11).

FGF21 is considered a typical fasting hormone and although seemingly paradoxical, circulating FGF21 levels are also elevated during obesity (12). The link between obesity and prolonged fasting is that they are both characterized by increased levels of circulating free fatty acids (FFAs), which can activate PPARα in the liver, leading to upregulation of FGF21 (13). In addition, FGF21 is secreted by the muscle during situations of metabolic stress [reviewed in Ref. (14)]. FGF21 acts on different tissues, including brain, adipose tissues, pancreas, and the liver [reviewed in Ref. (15)].

FGF1 is expressed in several tissues, including the liver, kidney, and brain, but most notably it is highly upregulated in white adipose tissue (WAT) following a high fat diet (HFD) challenge. Experiments with FGF1 KO mice revealed that this growth factor is indispensable for WAT remodeling in response to feeding and fasting. Mice lacking FGF1 are unable to properly expand their WAT during increased nutrient load, and upon withdrawal of the HFD, also have problems in WAT reduction (3).

When overexpressed or pharmacologically administered to obese, diabetic animals, FGF1, FGF19, and FGF21 all greatly improve the metabolic profile (4, 16, 17). Acute effects include lowering of blood glucose and insulin levels (16, 18). Chronic administration of any one of these FGFs results in increased insulin sensitivity, reduced hepatic steatosis (fatty liver), and improved serum lipid profiles. FGF15/19 and FGF21 also promote weight loss (18). FGF1, FGF19, and FGF21 regulate different metabolic processes through different cell types and tissues, of which the WAT, CNS, and the liver seem to be the main players (3, 4, 11, 14, 16, 18, 19). In addition, brown adipose tissue (BAT), pancreas, and muscle are involved in FGF signaling by being a source of FGFs, a target, or both (20–25).

FGF1, FGF15/19, FGF21, and their targets provide interesting therapeutic possibilities for the treatment of metabolic diseases, such as obesity, non-alcoholic fatty liver disease, type 2 diabetes, and atherosclerosis (3, 4, 17). Despite their spectacular metabolic properties, the native FGFs do not have the optimal characteristics to be used as a drug in the clinic (26, 27). However, considerable progress has been made in the development of highly improved FGF mutants and variants, one of which has already entered a clinical trial (28).

With this review, we aim to provide a comprehensive overview of the current concepts regarding FGF signaling in metabolic health and disease, and to provide starting points for the development of FGF-based therapies for metabolic diseases.

## FGF Signaling Machinery

The FGF family consists of 18 members affecting a variety of processes through induction of intracellular signaling via their cognate receptors, the FGFRs (29). There are four FGFRs (FGFR1–4) with an intracellular tyrosine kinase domain, and one that lacks this domain (FGFRL1/FGFR5) (30). Depending on the tissue, alternative splicing of these genes gives rise to a total of seven different isoforms (FGFR1b, FGFR1c, FGFR2b, FGFR2c, FGFR3b, FGFR3c, and FGFR4) due to the alternative use of exon IIIb or IIIc (29).

FGF1 is often called the universal ligand as it can bind and activate all FGFRs (31). It does, however, require polysulfated polysaccharides such as heparan sulfate proteoglycans (HSPGs) or heparin on the cell membrane to form a stable signaling complex to induce efficient signaling (32). These polysaccharides are present on the cell membrane of almost all cell types. It is thought that the high affinity of FGF1 and other canonical FGFs for HSPGs restricts their activity to the vicinity of its secretory point, and that they therefore mainly act as autocrine or paracrine factors (33). The interaction of FGF1 with integrins, another type of cell surface receptor, also contributes to its activity (34, 35).

FGF15/19 and FGF21 have lower affinities for HSPGs and thus can circulate through the body. They rely on the membrane-bound co-receptor β-klotho to establish FGFR activation (36, 37). Depending on the secreting tissue, FGF15/19 and FGF21 can act as endocrine and/or autocrine factors. FGF21 mainly binds to FGFR1, while FGF19 associates with both FGFR1 and FGFR4 to a similar extent (38). The tissue-specific activity of an FGF is thus determined by its affinity for the different FGFRs together with its requirement for binding to polysulfated polysaccharides, β-klotho and integrins (39).

Binding of an FGF to its receptors and cofactors induces dimerization and subsequent phosphorylation of the receptor intracellular tyrosine kinase domains, which then function as docking sites for other signaling proteins (40). Two main signaling proteins that associate with FGFRs upon receptor activation are phospholipase C (PLCγ) (41) and FGF receptor substrate 2 (FRS2) (42). PLCγ links FGFR activation to downstream changes in diacylglycerol (DAG), inositol triphosphate (IP3), intracellular Ca^2+^ levels, and activation of protein kinase Cs (PKCs). FRS2 facilitates the assembly of a scaffold complex [consisting of protein tyrosine phosphatase, non-receptor type 11 (Shp2), growth factor receptor-bound protein 2 (Grb2), and GRB2-associated-binding protein 1 (GAB1)] that mediates Ras/MAPK/ERK and PI3K–Akt signaling (31, 40, 43). Other studies showed that FGFRs can stimulate STAT3 activation (44). FGF-induced downstream signaling pathways and concomitant intracellular changes are tissue and cell type dependent.

The expression of β-klotho, FGFR1c, and FGFR2c is downregulated in WAT during obesity and inflammation (45). Pancreatic β-klotho is downregulated under hyperglycemic conditions (46). This indicates that FGF signaling is not only regulated by the time- and tissue-dependent expression of the ligands but also by their signaling machinery, which can be affected by pathological conditions. This further substantiates the intricate interplay between FGFs, receptors, and cofactors and their effect on metabolic homeostasis.

## FGF Signaling in White Adipose Tissue

**Figure 1:**
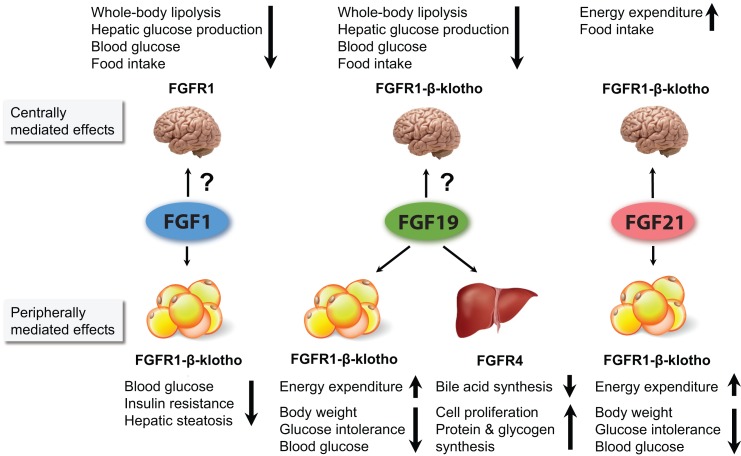
**Figure summarizing the major metabolic effects of pharmacological administration of FGF1, FGF19, and FGF21**. FGF1 and FGF19 can affect metabolism when infused directly into the brain. However, it is currently unclear to what extent they cross the BBB when peripherally administered, and whether the concentration that reaches the brain influences whole body physiology. For FGF21, it has been shown that its central actions contribute considerably to its pharmacological effects. All three FGFs are thought to affect whole body metabolism via signaling in the WAT. In addition, FGF19 also acts via the liver.

Adipocytes express both β-klotho and FGFRs (mainly FGFR1c and FGFR2c) and are therefore putative targets for FGFs, as illustrated in Figure 1 (47). WAT is also a source of FGFs, of which FGF1 and FGF21 are most relevant in the adult (3, 48). The adipose-tissue-specific FGFR1 KO mouse model has been particularly useful in unraveling the contribution of WAT to the beneficial effects of FGFs on metabolism. However, these mice were generated by Cre-recombinase-mediated deletion using the aP2 promoter, which is known for its ectopic expression in macrophages and neuronal cells (49). Several brain areas also express FGFR1 and have been shown to be involved in regulating responses to FGFs (50, 51). It is unknown whether there are neurons that co-express aP2 and FGFR1, which would result in excision of FGFR1, and defective FGFR1 signaling in the brain. Therefore, conclusions based on this model should be drawn with some caution.

### FGF1

Compared to lean controls, obese patients display a higher subcutaneous WAT-specific secretion of FGF1, as determined by *ex vivo* secretion assays (52). Adipose-derived FGF1 does not enter the circulation, suggesting that it acts locally (52). In mice, HFD feeding induces PPARγ-regulated FGF1 expression in visceral WAT, where it regulates responses to nutrient fluctuations. Knockout of FGF1 results in a defective response to HFD feeding characterized by aberrant WAT expansion and impaired WAT vascularization, the rapid development of severe diabetes, and defective WAT reduction upon HFD withdrawal (3). In contrast, FGF1 KO mice maintained on a normal chow diet show no obvious phenotype and appear completely normal.

How FGF1 is involved in the expansion of adipose tissue during HFD feeding is not completely understood. However, there is evidence that FGF1 promotes pre-adipocyte proliferation and differentiation, and that ERK1/2 signaling is central to these processes (53, 54). In addition, since FGF1 promotes angiogenesis, it is likely that locally produced FGF1 contributes to the expansion of WAT by stimulating vascularization (55). This hypothesis is supported by the finding that FGF1 KO mice show reduced vascularization of WAT after HFD feeding compared to control animals (3).

FGF1 is downstream of PPARγ, a well-known target for the thiazolidinedione (TZD) class of anti-diabetic drugs, leading to the hypothesis that FGF1 mediates a subset of PPARγ-activated genes, and by doing so improves the metabolic profile. Indeed, it has been shown that pharmacological administration of FGF1 normalized blood glucose levels within an hour in obese, diabetic rodents. Chronic administration resulted in normoglycemia, insulin sensitization, and reduced hepatosteatosis (fatty liver). The acute blood glucose lowering effect seems to be dependent on FGFR1 signaling in WAT, since WAT-specific deletion of this factor abrogated FGF1-induced normoglycemia in obese, hyperglycemic mice. Whether the other metabolic improvements (i.e., reduction of hepatic steatosis) are dependent on WAT signaling is unknown.

### FGF19

FGF19 has high affinity for both FGFR1 and FGFR4 (38). It needs β-klotho to induce signaling through FGFR1c, 2c, and 3c but can signal through FGFR4 independent of β-klotho (56). WAT is a putative target for FGF19, although it is believed that under normal physiological conditions the effects of endogenous FGF15/FGF19 are mainly mediated by the liver (5, 11, 17, 18). Pharmacological administration of FGF19 to obese diabetic rodents elicits beneficial effects on the metabolism that are highly similar to FGF21 administration (57).

In WAT-specific FGFR1 KO mice, FGF19 treatment still induced signaling in adipose tissue, and the beneficial effects on blood glucose levels were preserved. This might be explained by the fact that FGFR4 was upregulated in these mice, which possibly compensated for the loss of FGFR1 in the WAT (18). Along the same line, it was shown in *ob/ob* mice that an FGF19 variant (FGF19dCTD) that could not interact with FGFR1–3 but retained the ability to bind and activate FGFR4 was able to induce signaling in the liver but not in WAT. This mutant repressed CYP7A1 in the liver (which is a hallmark effect of hepatic FGF19 signaling) but failed to improve blood glucose levels in *ob/ob* mice (56). In accordance, another FGF19 variant with reduced affinity for FGFR4 but not FGFR1c was able to maintain its anti-diabetic effects (58). Finally, deletion of FGFR4 in mice impaired FGF19-mediated bile acid regulation but not its effects on improvement of glucose tolerance (58). Together, these data indicate that the pharmacological effects of FGF19 rely on WAT signaling (mainly through FGFR1/β-klotho) to improve blood glucose homeostasis. It is very likely that FGF19 induces the same intracellular signaling as FGF21. Indeed, it has been shown that injection of FGF19 induced similar patterns in gene expression as compared to FGF21 (58). It is less clear whether the other beneficial effects of FGF19 on the obese metabolic profile (i.e., increased energy expenditure and weight loss after chronic treatment, improved lipid profile) are also mediated via WAT, or whether this requires additional signaling in other metabolic tissues such as liver. However, because FGF19 stimulates proliferation in the liver it fell out of fashion as a putative drug to treat metabolic diseases (26).

### FGF21

In contrast to FGF1, which mainly acts at the site of its production, FGF15/19 and FGF21 not only act locally but can also escape into the circulation. FGF21 is mainly produced by the liver during times of metabolic stress, such as prolonged fasting (14). It is thought that one of the main targets is WAT, where FGF21 regulates aspects of the fasting response (10). Studies with WAT-specific FGFR1 KO mice indicate that FGF21 signaling through FGFR1/β-klotho in WAT protects the liver against steatosis during prolonged fasting by curbing lipolysis in WAT (59). In contrast, similar experiments in FGF21-overexpressing mice showed that FGF21 induces lipolysis in the WAT (10). These differences might be explained by compensatory regulation in knockout and transgenic models, non-specific effects by supra-physiological FGF21 levels or by other experimental differences. Therefore, the effects of FGF21 on lipid metabolism in WAT during prolonged fasting remain elusive. In addition, it was shown that, similar to FGF1, FGF21 expression was induced in the WAT upon feeding, where it stimulated PPARγ activity and locally aids in adipogenesis (48).

Most data on FGF21 signaling in WAT come from experiments in which FGF21 is pharmacologically administered or overexpressed in obese rodents, revealing potent effects on the amelioration of the metabolic profile during obesity (4, 60). Beneficial effects include lowering of plasma glucose, insulin, FFA and triglycerides, weight loss, and increased energy expenditure (4, 18, 60). In addition, FGF21 induces secretion of adiponectin, which was shown to be responsible for many of the effects of FGF21 in obese rodents. Studies in adiponectin-deficient animals showed that in these mice the acute beneficial effects of FGF21 are severely blunted, as are many of the chronic effects. Interestingly, the decrease in fat mass and body weight upon FGF21 treatment was still present in adiponectin-KO mice (albeit less than in FGF21-treated obese WT mice), indicating that these effects are independent of adiponectin (61, 62).

Signaling through the FGFR1/β-klotho complex is essential for many of the metabolic improvements that are induced upon FGF21 injection or overexpression in obese animals. KO or knockdown of β-klotho results in the ablation of FGF21 signaling in WAT, both *in vitro* and *in vivo*, and eliminates its beneficial effects in obese rodents (37, 47, 63, 64). WAT-specific FGFR1 KO results in similar ablation of FGF21 signaling. In these mice, FGF21 treatment failed to reduce plasma glucose, insulin, and triglycerides, and no longer stimulated the secretion of adiponectin (18). The beneficial effects of FGF21 on serum FFAs and the reduction in hepatic steatosis, however, were still present (18). Together, these findings indicate that the glycemic effects are mediated via FGFR1/β-klotho signaling in WAT, while the improvement in serum FFAs and hepatic steatosis is possibly regulated via direct FGF signaling in the liver and/or brain.

Experiments in liver-specific insulin-receptor knockout mice (LIRKO mice) showed that FGF21 improved whole body insulin sensitivity and reversed hyperglycemia independent of insulin sensitization in the liver. On the other hand, intact hepatic insulin signaling was required for the FGF21-mediated improvement of circulating cholesterol and the reduction of hepatic triglycerides (65).

The intracellular signaling pathways conveying the FGF21-induced metabolic changes in WAT are not completely understood. The phosphorylation of FRS2α upon FGF21 treatment has been well established, both *in vitro* and *in vivo*. Also, strong transient activation of ERK1/2 has been reported in several different studies (4, 37, 38, 57, 66). In 3T3-L1 adipocytes, FGF21 acutely stimulated transient phosphorylation of Akt, phosphorylation of GSK3, SHP-2, P70S6K, STAT3, Raf-1, and induced calcium fluxes (66). Other acute phosphorylation events were found in pathways involved in insulin receptor signaling and the Phospholipase C signaling pathway (67). It has been hypothesized that the rapid blood glucose lowering after FGF21 administration is regulated by an ERK-dependent, Elk-1- and SRF-mediated increase in GLUT1 expression in WAT (4, 68). However, the lowering of blood glucose by FGF21 *in vivo* is already apparent after 15 min, which is too fast for a process under transcriptional control (18). This indicates that other more acute signaling processes must be involved.

With respect to gene expression, a single FGF21 injection induced many changes in WAT, including changes in genes involved in FGF signaling, Wnt/β-catenin signaling, glucose uptake, amino acid transport, fatty acid oxidation, and lipid metabolism (60, 67). One of the main effects of chronic FGF21 treatment is increased energy expenditure (60). It has been proposed that in WAT, this process this is mediated by LKB1, a protein kinase upstream of the major metabolic regulator AMPK. FGF21 activates LKB1, which activates AMPK by phosphorylation of Thr172. This leads to activation of Sirtuin 1 (SIRT1), which in turn switches on several other metabolic pathways that lead to increased energy expenditure (69).

It remains difficult to differentiate the long-term effects and gene expression data from direct signaling events in the adipose tissue, and to differentiate between primary effects of FGF21 on WAT, and secondary effects mediated by downstream effectors (such as adiponectin), FGF signaling in other tissues, and by responses to shifts in whole body metabolism.

## The Effects of FGFS on Brown Adipose Tissue and Browning of WAT

The main function of white adipocytes is to store energy, and brown adipocytes primarily burn energy and produce heat as a net result. They do so by converting chemical energy into heat through expression of uncoupling protein 1 (UCP1) in the mitochondrial membrane to uncouple substrate oxidation from ATP synthesis, thereby generating heat. BAT has gained increased attention as a putative target for the treatment of obesity and T2D due to its “fat-burning” properties and the recent demonstration that it is present in adult humans (70).

Evidence is also accumulating that FGF signaling is involved in the regulation of BAT and browning of WAT. BAT expresses FGFR1 and β-klotho but is also a source of FGF21. Upon cold exposure or β-adrenergic stimulation (classical stimuli for BAT activation), the expression and secretion of FGF21 from BAT is induced (29, 71–73). Based on these findings, a regulatory mechanism has been proposed in which norepinephrine, released from the sympathetic nervous system in response to cold, increases cAMP levels by acting on β-adrenergic receptors in BAT. cAMP in turn activates PKA and p38 MAPK, which increase binding of the transcription factor ATF2 to the FGF21 promoter, thereby induce FGF21 gene transcription (73). BAT-derived FGF21 can act locally, or escape into the circulation to act on other tissues, and thus functions as an autocrine and endocrine factor involved in thermogenesis and responses to cold exposure. This is further supported by the observation that FGF21 KO mice display an impaired response to cold exposure (72). Another observation that stresses the importance of FGF21 in thermogenesis is that hepatic activation of PPARα by fatty acids from milk and subsequent induction of FGF21 is crucial for adequate BAT activation and thermogenesis in newborn mice (74).

Pharmacological FGF21 treatment or overexpression leads to an increase in expression of several genes and processes involved in thermogenesis in BAT. These include upregulation of UCP1 and Acetyl CoA Carboxylase 2 (ACC2), increased core body temperature, and increased glucose uptake into BAT (60, 65, 72, 75). In addition, FGF21 has also been shown to stimulate the expression of thermogenic genes and BAT markers (such as CIDEA and Cox7A) in WAT. The so-called “browning” of WAT is characterized by the appearance of brown-like adipocytes in WAT, a process normally induced by cold or β-adrenergic stimulation (67, 72, 76, 77). PGC-1α, which is an important regulator of mitochondrial function, oxidative metabolism and thermogenesis, is crucial in the thermogenic effects of FGF21 on WAT and BAT. Knockout of PGC-1α greatly impaired the expression of thermogenic genes in response to FGF21 (72). *In vitro* treatment of isolated primary WAT and BAT adipocytes with FGF21 induced thermogenic gene expression (e.g., UCP1, PGC1-α, and cytochrome *c*). Unlike many other FGF-mediated processes, the FGF21-induced thermogenic gene expression seems to be ERK independent, since it was not impaired by addition of an ERK inhibitor to BAT cell cultures (72).

FGF21 does not acutely stimulate glucose uptake into brown adipocytes *in vitro* but is able to augment insulin-stimulated glucose uptake (78). Prolonged (24 h) treatment of cultured brown adipocytes with FGF21 increased total respiration, uncoupled respiration, and glucose consumption (73). This indicates that FGF21 can directly act on BAT and the browning of WAT, and suggests a positive feedback loop *in vivo*, where cold exposure leads to increased FGF21 expression and secretion, mediating upregulation of the thermogenic program. However, the effects of FGF21 on thermogenesis by regulating the nervous system cannot be excluded, since it has been shown that FGF21 also affects energy homeostasis via the brain, which in turn regulates neuronal output to peripheral organs (50, 51, 72, 73).

The finding that FGF21 directly activates the thermogenic program in BAT and WAT, and increases energy consumption *in vitro*, suggested that its beneficial pharmacological effects in obese rodents might be mediated by BAT. However, this hypothesis has been severely challenged. Two groups independently showed that surgical removal of BAT does not impair the pharmacological actions of FGF21 in obese rodents (79, 80). In addition, Véniant and colleagues showed that FGF21 retained most of its beneficial effects in UCP1 KO mice, indicating that BAT activation and WAT browning alone cannot account for the improved metabolic parameters upon FGF21 treatment (81). Similar observations were done by Samms et al. (82), who showed that UCP1 KO mice still displayed improved glycemic control and improved lipid profiles, and showed weight loss mainly due to decreased food intake. A recent study by Kwon and colleagues suggested that reductions in body weight upon FGF21 treatment is independent of UCP1, agreeing with the previous studies. However, their data suggest that FGF21-dependent glucose clearance requires UCP1 protein (83).

In summary, several lines of evidence indicate that FGF21 plays an important role in the physiological response to cold exposure by acting on the BAT. The notion that BAT increases FGF21 expression upon cold exposure implies that there is a local feedback loop. In addition, since FGF21 also has endocrine effects, it cannot be excluded that BAT-derived FGF21 acts on other organs (i.e., brain and WAT) contributing to the cold response. However, there is substantial evidence that the pharmacological effects of FGF21 are not dependent on BAT but instead rely on other tissues such as the brain and WAT.

## Hepatic FGF Signaling

Abundant expression of FGFR4 and β-klotho makes the liver an important target for FGFs in both mice and humans, as illustrated in Figure 1 (38, 84, 85). Next to WAT, the liver is the most investigated target and source of FGFs in the context of FGF signaling in metabolic homeostasis.

### FGF19

The first indication that FGFs play a role in hepatic energy metabolism came from FGFR4 knockout mice which displayed increased bile acid production (86). Bile acids play an important role in nutrient absorption but can also function as signaling molecules through activation of bile acid receptors FXR and TGR5 (87). The discovery that postprandial production of FGF15/19 in the ileum is transcriptionally controlled by FXR provided an essential lead toward elucidating the regulatory mechanism of bile acid homeostasis (88). FGF15/19 was shown to negatively regulate bile acid production by activation of FGFR4/β-klotho on hepatocytes and subsequent suppression of CYP7A1 and CYP8B1, genes that encode the rate-limiting enzymes in bile acid synthesis (88). This FGF19-induced repression required both the activation of the c-Jun N-terminal kinase (JNK) and the ERK signaling pathway (89).

Injection or overexpression of FGF19 lowered blood glucose levels and triglycerides in diabetic mice, and protected against diet-induced obesity (5, 17). Conversely, KO of FGFR4, the preferential receptor for FGF15/19, caused hyperlipidemia, glucose intolerance, and insulin resistance. Surprisingly, however, FGFR4 KO mice were protected against HFD-induced hepatic steatosis. The same study showed by re-expressing hepatic FGFR4 in FGFR4 KO mice that FGFR4 in the liver was indispensable for whole body lipid metabolism but not required for whole body glucose metabolism (90).

Kir and colleagues showed that FGF15/19 does affect hepatic glucose metabolism by acting as an insulin-independent stimulator for protein and glycogen synthesis (11). Unlike insulin, which predominantly relies on the Akt–mTOR axis to influence these processes, i.v. injection of FGF19-stimulated protein and glycogen synthesis via FGFR4–ERK–RSK signaling. This in turn resulted in repression of the α- and β-isoforms of GSK3, de-repression of glycogen synthase, and enhanced glycogen storage. The same pathway also resulted in activation of eukaryotic initiation factors eIF4B and ribosomal protein S6 by RSK, leading to stimulation of protein synthesis in HepG2 cells as well as in mice (11).

Inhibition of the insulin-induced signaling cascade during insulin resistance occurs to a large extent upstream of Akt. FGF signaling therefore bypasses this upstream inhibition, while downstream processes such as glycogen and protein synthesis are activated, thereby mimicking insulin action. Insulin does not only stimulate glycogenesis but also promote lipogenesis through activation of sterol regulatory element-binding protein 1c (SREBP-1c), and repression of lipolysis and mitochondrial function through inhibition of the transcription factor forkhead box O1 (FOXO1), which causes hepatic triglyceride (TG) accumulation (91, 92). This steatotic effect was not observed in FGF19-treated mice. Finally, FGF19-induced ERK signaling was linked to repression of apolipoprotein A (APOA) in the liver, which is associated with athero-thrombotic disease (93).

A major finding regarding FGF signaling in the liver is that FGFR4 is responsible for FGF19-induced proliferation of hepatocytes and the induction of hepatocarcinomas (94). The observation that FGFR4 is involved in the regulation of proliferation led to the development of FGF variants that do not activate this receptor, so as to avoid mitogenesis (16, 26). This will be further discussed in the section about FGF mutants.

The upregulation of endogenously produced FGF15/19 may offer an alternative route to improve metabolism. It has been shown that gut-specific activation of FXR leads to increased FGF15 levels in mice and alterations in the bile acid plasma pool. Intestinal FXR activation did not lead to weight loss in obese mice but did improve several metabolic parameters. It is, however, unclear which of these effects are attributable to FGF15, and which ones to the changes in bile acid composition. In addition, it is unclear whether systemic upregulation of FGF15 in this model leads to increased hepatocarcinomas (95).

Together, these findings indicate that hepatic FGF19 signaling not only affects bile acid synthesis but also influences many other aspects of energy metabolism. Which receptors mediate these different responses is still unclear and is probably determined by the physiological state of the animal (i.e., normal body weight or obese, fed or fasted) and by the route of administration (i.e., overexpression, injection).

### FGF21

The liver is an important source of FGF21. Experiments in mice with hepatic FGF21 overexpression suggested that FGF21 induces lipolysis in WAT, ketogenesis in the liver, reduction of physical activity, and induction of torpor (10). These are all aspects of a starvation response, in which the organism shifts from carbohydrates as an energy source to utilization of fatty acids released from the WAT, and in which ketone bodies are produced that supply the brain with energy. Based on the finding that mice with a liver-specific deletion of FGF21 displayed reduced peripheral insulin sensitivity, it has been hypothesized that upon refeeding after prolonged fasting, hepatic FGF21 communicates with other tissues to induce adequate refeeding responses, thereby helps in overcoming fasting-associated peripheral insulin-resistance (78).

Acute treatment of wild-type C57Bl/6 mice with FGF21-induced hepatic FGF signaling, marked by FRS2α and ERK phosphorylation, and the induction of genes involved in hepatic gluconeogenesis, lipid metabolism, and ketogenesis (96). Chronic treatment of obese rodents with FGF21 reduced hepatic steatosis and improved insulin sensitivity, indicating that FGF21 treatment also improves liver function (75). These changes were associated with reductions of SREBP-1 in the nucleus, and concomitant downregulation of several of its target genes, including genes involved in hepatic glycolysis, *de novo* fatty acid synthesis, and triglyceride synthesis. Repression of glucose-6-phosphatase was also found, suggesting repression of glycogenolysis (75).

It remains elusive how FGF21 mediates changes in the liver, and whether its effects are direct or indirect. The liver mainly expresses FGFR4, and only low levels of FGFR1, the preferential receptor for FGF21 (29, 38). FGF21 is unable to establish signaling through the FGFR4-β-klotho complex (38). Therefore, it remains to be determined whether the low levels of FGFR1 are sufficient to induce FGF21 signaling, or whether, for example, FGFR2 and FGFR3, which are expressed at low levels in the liver, under certain circumstances might also contribute to hepatic FGF21 signaling (29, 38). In addition, it is possible that the changes in hepatic metabolism in response to chronic FGF21 treatment are a consequence of a generally improved whole body metabolism, rather than a direct action of FGF21 on the liver.

## Fibroblast Growth Factors and Metabolic Regulation via the Central Nervous System

It is well-established that peripheral signals such as adipokines and gastro-intestinal hormones (e.g., leptin and cholecystokinin) convey metabolic information to the brain and dynamically modulate the neuronal regulation of energy intake and glucose homeostasis (97). Disturbances or defects in this neuronal regulatory system of energy metabolism therefore often contribute to the development of obesity, metabolic syndrome, and type 2 diabetes (98).

FGF1, β-klotho, and FGFRs are differentially expressed in several parts of the central nervous system (29, 99, 100). So far, no expression of FGF19 or FGF21 has been found in the brain (50), but it has been shown that FGF21 can easily enter the brain by diffusion, while FGF15/19 seems to lack this property and mainly stays in the peripheral circulation (101, 102).

Several lines of evidence now support a neuro-modulatory role for FGFs and their receptors in the central regulation of food intake, glucose homeostasis, and circadian behavior, and in mediating at least some of the pharmacological effects of FGFs (Figure 1). The recent finding that FGFR1 and FGFR4 expression in the rat hypothalamus are dramatically reduced in response to HFD further underlines the involvement of the FGF–FGFR axis in the central regulation of metabolism (100).

### FGF1

FGF1 was the first member of the FGF family demonstrated to play a role in the neuronal regulation of food intake. Early studies in rats showed that feeding or intraperitoneal glucose injections increased FGF1 concentration in the cerebrospinal fluid. Ependymal cells lining the third cerebral ventricular wall are considered to be the primary source of this FGF1 (99). Subsequently, it was demonstrated that intra-cerebro-ventricular (i.c.v.) infusions of FGF1 suppressed food intake and inhibited the activity of FGFR1-containing glucose-sensitive neurons in the lateral hypothalamus (LHA), probably by activating PKC (25, 99, 103). Conversely, neutralization of the biological activity of FGF1 by infusion of anti-FGF1 and/or FGFR1 antibodies into the LHA increased food intake (104, 105).

The generation of different FGF1 peptide fragments showed that the amino-terminal (1–15) of the molecule, but not the carboxyl-terminal, is responsible for the anorexic effect of FGF1 (106). FGF1-induced feeding suppression is also strongly associated with the selective induction of heat shock protein 27 (HSP27) in hypothalamic astrocytes surrounding the third ventricle (107). However, the physiological relevance of increased HSP27 expression and the role in FGF1-induced suppression of feeding behavior remains unclear. In addition to its effects on feeding, it has also been shown that i.c.v. infusion of FGF1 in rats resulted in a dramatic increase in slow wave sleep (i.e., deep sleep), which is considered a behavioral display of satiety that follows feeding (108). Although the effects of centrally administered FGF1 on food intake are quite clear, its effect on body composition and energy expenditure remain to be investigated. In addition, it is unknown whether peripherally administered FGF1 can cross the blood–brain barrier, and therefore whether the pharmacological effects of FGF1 are partly attributable to signaling in the brain.

### FGF19

The presence of FGFR4 and β-klotho in the hypothalamus suggested that part of the metabolic actions of FGF19 could be mediated via the brain (100). Indeed, delivery of small doses of FGF19 directly into the brain has been shown to increase energy expenditure in mice (17). Moreover, i.c.v. infusion of FGF19 reduced food intake and acutely improved glucose tolerance in both lean and diet-induced obese rats. Opposite effects on food intake and glucose tolerance were found when FGF signaling was blocked by central delivery of the FGFR inhibitor PD173074 (100). Another study using *ob/ob* mice as a model supported these findings and suggested that a single i.c.v. infusion of FGF19 improved glucose intolerance by increasing glucose disposal rate (24). In a rat model of type 1 diabetes, it was further shown that i.c.v. infusion of both FGF1 and FGF19 suppressed the hypothalamic–pituitary–adrenal (HPA) axis, resulting in a decrease in hepatic glucose production, hepatic acetyl CoA content and whole-body lipolysis (109). However, a study on FGF19 kinetics showed that FGF19 enters the brain with low efficiency, and that after peripheral administration of supra-physiological doses only a small fraction enters the brain (102). It is therefore believed that both endogenously produced FGF15/19 and pharmacologically administered FGF19 mainly function via the peripheral tissues and not via the CNS.

### FGF21

FGF21 is not expressed within the CNS but can enter the brain relatively easily, which allows for communication between peripheral tissues and the CNS (23).

The β-klotho^Camk2a^ mouse, which lacks β-klotho expression in the hypothalamus and the hindbrain, has been instrumental in teasing out the central effects of FGF21 as opposed to its peripheral effects. Using this model, it was shown that, during normal physiology, central FGF21 signaling is required for the regulation of circadian rhythms and the adaptive starvation response, which includes suppression of female reproduction and physical activity and the systemic increase of glucocorticoids (50). The central player in these processes is the hypothalamus, where FGF21 signaling suppressed arginine vasopressin (AVP), and stimulates corticotropin-releasing factor (CRF). Activation of the CRF–ACTH axis ultimately leads to adrenal glucocorticoid secretion, which in turn stimulates hepatic gluconeogenesis, thus providing an FGF21-mediated communication loop between the liver and the brain (50, 110). CRF can also activate BAT via sympathetic nervous output (111).

FGF21 signaling in the central nervous system has also been shown to be important for the beneficial effects of FGF21 in obese rodents. The first evidence came from experiments in which FGF21 was centrally infused into obese rats. This resulted in increased food intake, energy expenditure, and insulin sensitivity due to increased insulin-induced suppression of both hepatic glucose production and gluconeogenic gene expression, but without changes in glucose utilization (51). Experiments with HFD-fed β-klotho^Camk2a^ mice showed that β-klotho in the hypothalamus is needed to recapitulate the majority of the beneficial effects of FGF21 treatment during obesity. In obese, hypothalamic-deficient β-klotho mice, the increase in energy expenditure, decrease in body weight, and subsequent improved changes in metabolic parameters, which normally occur upon chronic FGF21 administration, were all abrogated (19). This pointed toward the CNS as an important target for FGF21, both during normal physiology and during pharmacological treatment.

In conclusion, FGFs are able to affect the brain and modulate food intake and energy expenditure both during normal physiology and in animal models of obesity. The brain should therefore be considered as a potential target of these growth factors in the treatment of metabolic diseases.

## FGF Signaling in the Adult Pancreas

Although FGFs and FGFRs have long been known to play an important role in the embryonic development of the pancreas; more recently, it has been demonstrated that they also have a function in the adult pancreas (21, 112–114). In mice, the adult pancreas expresses β-klotho, moderate levels of FGFR1, but virtually undetectable levels of the other three conventional FGFRs (29). The expression of FGF21 in pancreatic islets and isolated rat β-cells is well established (21, 29). The expression of other FGFs, however, is controversial. Some studies report the presence of FGF1, FGF2, FGF4, FGF5, FGF7, and FGF10 in β-cells (113), whereas others did not observe the expression of these factors (29).

Pancreatic FGF signaling has been linked to β-cell function by the discovery that disruption of FGFR1c-induced signaling in the pancreas accelerates the development of diabetes (113). Overexpression of a dominant-negative FGFR1c receptor in the pancreas caused β-cell dysfunction, decreased β-cell population, impaired expression of glucose transporter 2 (GLUT2), and aberrant insulin processing.

So far, the effects of FGF21–FGFR1 in β-cells and its effects on insulin secretion received the most attention because of its possible relevance to diabetes. Less well-studied is how FGF21 injection or FGF21 incubation of islets works on α-cells and leads to the reported lowering in glucagon levels and glucagon secretion (4). In islets from non-obese, non-hyperglycemic rats, FGF21 has been shown to inhibit glucagon secretion but not insulin secretion (4). In another study, it was found that in islets from diabetic rodents FGF21 increased insulin content and secretion, and that FGF21 partially protected the islets from glucolipotoxicity and cytokine-induced apoptosis through ERK1/2- and Akt-dependent signaling mechanisms (21). In contrast, in islets isolated from healthy rats, FGF21 only increased insulin mRNA and protein levels but did not potentiate glucose-induced insulin secretion (21). Both in Ins-1E cells and in isolated rat islets, FGF21 treatment resulted in phosphorylation of FRS2α, Akt, and ERK1/2. The increase in insulin content upon FGF21 treatment was ERK1/2 dependent, while the increased survival was suggested to be mediated via Akt and activation of BAD (21).

Another mechanism by which FGF21 could protect against HFD-induced metabolic stress in β-cells is via the repression of Acetyl-CoA carboxylase (ACC). ACC is the rate limiting enzyme for the conversion of citrate into malonyl-CoA, a substrate for long-chain fatty acid synthesis and potent inhibitor of mitochondrial transport and oxidation of fatty acids, two processes that contribute to β-cell malfunction (115). FGF21-treated islets showed decreased ACC expression and were protected against palmitate-induced toxicity (116).

A non-canonical FGF receptor, designated FGF receptor 5 or FGF receptor-like 1 (FGFR5, FGFRL1) was also found to be expressed in the adult pancreas (117). FGFR5 localizes to the plasma membrane and to the insulin secretory granules (118). Unlike the canonical FGFRs, FGFR5 lacks an intracellular kinase domain and has therefore been postulated to function as a decoy receptor. Despite this, FGFR5 overexpression resulted in a ligand-independent phosphorylation of ERK1/2, which was found to be dependent on the presence of the Src homology domain-2 (SH2)-binding motif and the histidine-rich region in the C-terminal part of the receptor. Based on these findings, it has been proposed that binding of SHP-1 phosphatase to the SH2 domain leads to the ligand-independent activation of ERK1/2. FGFR5 overexpression in β-cells resulted in increased insulin content and secretion, and increased extracellular matrix adhesion. No effect, however, was observed on proliferation (118).

## FGF Signaling in Muscle

A role for FGFs in skeletal muscle regeneration was first described for FGF6, which is restricted to the myogenic lineage (119). FGF6-deficient mice show severe skeletal muscle regeneration defects characterized by fibrosis and myotube degeneration. It is believed that FGF6 primarily signals through FGFR4, since this receptor is highly expressed during myoblast differentiation and in recently formed myotubes (119, 120). Adult skeletal muscle tissue, on the other hand, only expresses moderate levels of FGFR1, low levels of β-klotho, and very low to undetectable levels of the other FGFRs (29, 85). The heart also expresses FGFR1 and low levels of β-klotho. Therefore, the skeletal muscle and heart are putative targets for FGFs in the adult.

It is unclear whether FGFs can influence energy homeostasis by directly acting on skeletal muscle tissue. Some studies report an improvement of glucose uptake into the muscle after FGF21 injection in obese mice (which could be a direct or an indirect effect), while others did not find this (75, 121). In human cultured myotubes, it has been found that FGF21 protected against palmitate-induced insulin resistance by affecting NF-κB signaling (122). In addition, FGF21 has been reported to stimulate glucose uptake in human cultured myotubes and mouse EDL muscle, but the signaling events involved in this process are unknown (22).

Although it remains unclear whether adult skeletal muscle is a direct target for FGFs in the context of metabolism, it is clear that it can be a source of FGF21. FGF21 protein was detected in muscle of fasted mice and was upregulated in gastrocnemius muscle of skeletal muscle-specific Akt1-transgenic mice (123). Increased expression of FGF21 in skeletal muscle has also been described after exercise and in various pathological conditions, such as hyperinsulinema, mitochondrial myopathy, and lipodystrophy (124–128). In addition, increased skeletal muscle expression and circulating levels of FGF21 were found in patients with iron–sulfur cluster deficiency (129). It has been hypothesized that muscle derived FGF21 acts as a stress signal on adipocytes to maintain metabolic homeostasis (14). Whether the muscle is involved in mediating the pharmacological effects of FGF21, FGF19, or FGF1 is unclear.

Similar to the skeletal muscle, the heart mainly expresses FGFR1 and low levels of β-klotho. Therapeutical treatment with either FGF1, FGF15/19, or FGF21 will therefore most likely also induce signaling in the heart. FGF2, which is very similar to FGF1, has been shown to affect cardiac remodeling through activation of MAPK [reviewed in Ref. (130)]. In addition, it has been shown that FGF21 KO mice are more susceptible to the development of cardiac hypertrophy also via activation of the MAPK signaling pathway (131). It is also possible that FGF21 signaling in the brain affects the sympathetic outflow to the heart, similar to BAT (19).

In conclusion, although the skeletal muscle and the heart can be targets for FGFs and are important for metabolic homeostasis, the effect of FGFs on these targets are largely unclear and poorly studied. However, proper assessment of the effects of FGF signaling on these targets (especially on the heart) is needed for the development of safe FGF-based drugs.

## FGF Mutants and FGFR Antibodies: Promising Perspectives for FGF-Based Pharmacological Treatments

The finding that FGFs play important roles in metabolic regulation offers new therapeutic options for the treatment of metabolic disorders. However, despite their spectacular effects in rodents, wild-type FGFs have several drawbacks for use in patients. They have various adverse effects, are expensive and labor-intensive to produce, not suitable for large-scale production, and/or have poor stability and short half-life, which currently holds back their application (16, 26, 27, 132, 133). To circumvent this, several attempts have been made to improve the FGFs, so that they are more stable, retain, or improve their beneficial effects on metabolism, and lose their side-effects.

The main concern that limits the application of FGF1 and FGF19 is that they have been linked to *in vitro* cellular proliferation and tumor formation, respectively (94, 134, 135). Although there are no indications that FGF21 is mitogenic, its chronic administration has been associated with bone loss (132). In addition, FGF1 and FGF21 have low *in vivo* stability, which would make treatment expensive and require multiple injections per week (27, 133). With regard to FGF1 and FGF19, attempts have been made to identify sequences within the peptide responsible for mediating the proliferative responses, and whether they can be deleted while retaining the metabolic properties. From studies with FGFR1 and FGFR4 KO mice and studies with FGF19 mutants that show differential binding preference toward FGFR1 and FGFR4 the concept arose that FGFR1 is the “metabolic” FGFR, while FGFR4 is the “proliferative” FGFR (18, 56, 58). This concept seems to be valid in many cases and has proved to be useful in the design of FGF variants with reduced mitogenic properties.

### FGF1 Mutants

**Figure 2:**
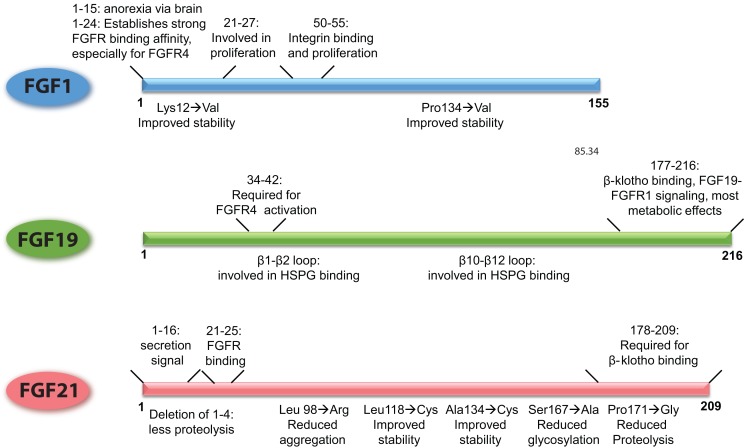
**Schematic overview of the FGFs, demarcating the regions of the proteins that underlie their functional properties**. In addition, it is shown how point mutations or small deletions affect the characteristics of that particular FGF. The figures are based on research data from Ref. (16, 34, 106, 133, 136) for FGF1 (56, 139) for FGF19, and from Ref. (27, 140, 141) for FGF21.

Already in the 90s, it has been shown that the N-terminus of FGF1 is involved in the proliferative properties of FGF1 [illustrated in Figure 2, Ref. (136)]. In addition, it has been shown that the N-terminus (amino acids 1–15) is important for the anorexic effects of FGF1 in the brain (106). More recently, the FGF1 variant FGF1dNT was described. This mutant lacks the first 24 amino acids from the N-terminus and shows reduced binding affinity for FGFR1 and 2, and virtually no affinity for FGFR3 and FGFR4. With the deletion of the N-terminus also the proliferative properties of FGF1dNT on the NIH 3T3 fibroblast cell line disappeared, while the *in vivo* insulin-sensitizing effects were conserved (16). Another FGF1 mutant, R50E no longer binds to integrin αvβ3 but can still bind to FGFR1 and heparin (34). Although it can still stimulate transient ERK phosphorylation, this activation cannot be sustained and consequently shows reduced effects on cell proliferation and cell migration (137). Moreover, this mutant was able to suppress the angiogenic and tumorigenic effects of FGF1 and FGF2 in several different *in vivo* and *in vitro* models (138).

As FGF1 can stimulate angiogenesis, it is currently under investigation for the treatment of ischemia and wound healing. However, its poor bio-stability currently limits its application. Attempts to improve the stability of the protein showed that changing a few amino acids within the protein (Lys12 → Val and Pro134 → Val) significantly improved the half-life and stability of FGF1 (133). Whether the FGF1 R50E mutant and the thermostabilized mutant hold promise for FGF1-based treatment of metabolic disease remains to be determined.

### FGF19 Mutants

FGF19 and FGF21 are structurally highly related, but FGF19 has proliferative effects and stimulates tumorigenesis in the liver, whereas FGF21 does not have these properties (94). Using FGF19/21 chimeric proteins, it has been shown that amino acid residues 38–42 of FGF19 are responsible for the activation of FGFR4 and proliferative effects on hepatocytes, a property that could be transferred to FGF21 (Figure 2). In addition, the β1–β2 loop and the β10–β12 loop are required for heparin induced FGF19–FGFR4 signaling, and also contribute to the mitogenic properties of FGF19 (139). Deletion of the C-terminus abolished β-klotho interaction, thereby FGF19-induced signaling through FGFR1c, 2c, and 3c but retained FGFR4 activation through heparin-mediated stabilization of the signaling complex. This FGF19 mutant could still suppress bile acid synthesis, whereas beneficial effects on glucose metabolism and insulin sensitivity were abrogated (139). In contrast, an FGF19 variant with a modified N-terminus was no longer able to affect bile acid metabolism, whereas glucose levels and insulin sensitivity could still be beneficially influenced. Interestingly, this variant also lost its proliferative properties, supporting the hypothesis that hepatocyte proliferation is dependent on activation of FGFR4 (26, 139).

### FGF21 Mutants

Of all the metabolic FGFs, FGF21 received the most attention because of its beneficial effects on metabolism, while at the same time lacking proliferative properties. Using C-terminal and N-terminal deletion mutants, it has been shown that, similar to FGF19, the C-terminus of FGF21 is required for β-klotho interaction, whereas the N-terminus drives efficient FGFR activation (Figure 2) (140, 142). Due to its poor stability and high production costs, most bioengineering research on FGF21 has been aimed at improving the stability and potency of the protein and bringing down the production costs. The results of this research have been recently extensively described (143, 144). In short, approaches to improve FGF21 bio-stability include the addition of an Fc fragment to the N-terminus, the introduction of non-native disulfide bonds and subsequent PEG-ylation, and conjugation of FGF21 to a scaffold antibody (141, 145, 146). The most successful FGF21 variant is LY2405319, which entered clinical trials (28). This FGF21 variant lacks the first four amino acids of the secretion signal, which reduces proteolysis at the N-terminus when the protein is expressed in yeast. The introduction of a non-native disulfide bond between Leu118 and Ala134 improved the stability of the peptide while substitution of Ser167 with an alanine reduced glycosylation and breakdown. Together, these modifications resulted in a protein that could be produced in yeast and is therefore suitable for large-scale production, is more stable, but retained the metabolic effects of native FGF21 with similar potency (27). This mutant successfully lowered body weight, improved blood lipid profiles and increased insulin sensitivity in obese human subjects with type 2 diabetes. Blood glucose levels on the other hand were not significantly lowered, although a trend was apparent (28). Some side-effects were reported, such as hypersensitivity, elevated liver enzymes, injection site reactions, and headache, but the vast majority of the test subject tolerated the treatment considerably well.

The aforementioned examples of the different FGF variants indicate that the proliferative and metabolic properties of these peptide hormones are located at different sites within the molecule and are also transduced by different receptors and cofactors. In addition, small modifications of the peptide can influence stability, purification, half-life, and production costs. Together, these options provide a window to tailor FGFs for specific pharmacological needs.

## Modulation of Metabolism Using FGFR Antibodies

In addition to modulation of FGFR signaling by modifying the ligands, it is also possible to target the FGFRs using specific activating or inhibiting antibodies. Antibodies that inhibit the proliferative FGFR-mediated signals have been extensively investigated for the development of anti-cancer drugs [reviewed in Ref. (147)]. More recently this approach has also been explored in the treatment of metabolic disease. An antibody (IMC-A1) that was originally developed for anti-proliferative therapy by inhibition of FGFR1 was found to effectively reduce food intake through FGFR1c inhibition in the hypothalamus (148). This is in contrast to an earlier study that reported increased food intake in response to an FGFR1-antibody, targeting FGFR1 in the hypothalamus (105).

Another interesting antibody that can improve metabolic dysfunction is the FGF21-mimetic antibody, R1MAb1, developed by Genentech (149). Although designed to mimic FGF21, this antibody activates the b and c splice variants of FGFR1 independent of β-klotho. It differs from FGF21 in these aspects, since FGF21 is β-klotho dependent and also activates FGFR2c and FGFR3c. Injection of R1MAb into obese mice had effects highly similar to FGF21 treatment, and included improvement of serum insulin, lipid, and blood glucose levels and increased energy expenditure. R1Mab stimulated ERK signaling in adipose tissue, but not in the liver (149).

A different FGF21 mimetic is mimAb1, developed by Amgen. This antibody only activates the FGFR1c splice variant, and is dependent on β-klotho to establish efficient signaling. Administration of mimAb1 to obese monkeys decreased body weight and improved several metabolic parameters, including plasma glucose and triglyceride levels. In addition, with a half-life of 11 days it is highly stable and long-acting. Since the metabolic effects of mimAb1 were not observed in WAT-specific FGFR1 KO mice, it was suggested that these effects are mainly mediated though the adipose tissue (150). A similar antibody (C3201-HSA) that has specific binding affinity for FGFR1 and human/monkey β-klotho was also developed by Amgen. This compound also greatly improved the metabolic profile of obese monkeys. Due to its high binding affinity for the human form of β-klotho, this antibody would be particularly suitable for use in human patients (151).

A slightly different FGFR1/β-klotho-activating antibody (bFKB1) also improved the metabolic profile of obese mice. Administration bFKB1 led to weight loss, BAT activation, and an overall improved metabolic profile but did not lead to elevated corticosterone levels. Interestingly, it did induce signaling in the adipose tissues and pancreas, but not in the liver and brain (152). Conversely, an antibody designed by AstraZeneca, R1c mAb, which also specifically targets FGFR1c, is thought to mainly exert its effects via the brain where it suppressed food intake (153). This indicates that both targeting the peripheral tissues and/or the brain can be successful in improving the metabolic status.

## Conclusion and Future Perspectives

The identification of several FGFs as hormones with high relevance to metabolic regulation has led to a revival of interest in this family. During normal physiology, FGF1, FGF15/19, and FGF21 have all been shown to regulate distinct but overlapping metabolic processes in a variety of metabolic cell types and tissues. The expression of FGFRs, cofactors and cell surface molecules and the affinity of different FGFs for these factors allow for this differential regulation. In addition, it has been shown that pharmacological administration to obese animals greatly improves their metabolic profiles and that WAT, liver and brain are important players in modulating these effects. Activation of FGFR1 seems central to modulating the beneficial metabolic effects.

Even though little is known about the intracellular events that underlie the metabolic responses to FGFs, great progress has been made in the development of FGF-based drugs. The use of chimeras of different FGFs has been instrumental in elucidating the functional roles of different domains in the FGFs and has resulted in greatly improved pharmaceutical properties (16, 34, 139). Targeting of FGFR1 with specific activating antibodies might be a viable approach to treat metabolic disease as well.

Within 10 years of its discovery as a metabolic hormone, FGF21 has made it from an obscure FGF to a serious candidate in the treatment of metabolic conditions. This holds promise for FGF1, of which its pharmacological properties were only recently discovered (16). In addition, it may be worthwhile to investigate whether individuals with defective FGF signaling are more prone to the development of metabolic aberrations, which might contribute to the development of more suitable treatments for these particular patients. We expect that further understanding of the tissues, receptors, and signaling pathways involved in the different metabolic improvements induced upon administration of FGFs and/or their pharmacologically improved variants will greatly aid in the development of safe, effective, and specific drugs for the treatment of metabolic disorders.

## Author Contributions

VN, GS, WL, TZ, DS, RY, AA, RE, JJ, and MD wrote the paper.

## Conflict of Interest Statement

The fibroblast growth factor (FGF) molecules and related methods of use are covered in the following published patent applications and counterparts that derive priority: (1) PCT/US2011/032848, held by Ronald M. Evans, Michael Robert Downes, and Johan W. Jonker (handled by Salk OTD); (2) PCT/US2013/044589, held by Ronald M. Evans and Michael Robert Downes (handled by NYU Office of Industrial Liaison/Technology Transfer); (3) PCT/US2013/044594, held by Ronald M. Evans and Michael Robert Downes (handled by NYU Office of Industrial Liaison/Technology Transfer). Michael Robert Downes and Ronald M. Evans are shareholders and consultants for the company Metacrine. The other co-authors declare that the research was conducted in the absence of any commercial or financial relationships that could be construed as a potential conflict of interest.
